# Temporal dynamics of a homeostatic pathway controlling neural network activity

**DOI:** 10.3389/fnmol.2013.00028

**Published:** 2013-09-18

**Authors:** Helen S. Bateup, Cassandra L. Denefrio, Caroline A. Johnson, Jessica L. Saulnier, Bernardo L. Sabatini

**Affiliations:** Department of Neurobiology, Howard Hughes Medical Institute, Harvard Medical SchoolBoston, MA, USA

**Keywords:** Arc, ERK signaling, TSC/mTOR, homeostatic plasticity, network activity, hippocampus, multi-electrode array, activity-dependent gene regulation

## Abstract

Neurons use a variety of mechanisms to homeostatically regulate neural network activity in order to maintain firing in a bounded range. One such process involves the bi-directional modulation of excitatory synaptic drive in response to chronic changes in network activity. Down-scaling of excitatory synapses in response to high activity requires Arc-dependent endocytosis of glutamate receptors. However, the temporal dynamics and signaling pathways regulating Arc during homeostatic plasticity are not well understood. Here we determine the relative contribution of transcriptional and translational control in the regulation of Arc, the signaling pathways responsible for the activity-dependent production of Arc, and the time course of these signaling events as they relate to the homeostatic adjustment of network activity in hippocampal neurons. We find that an ERK1/2-dependent transcriptional pathway active within 1–2 h of up-regulated network activity induces Arc leading to a restoration of network spiking rates within 12 h. Under basal and low activity conditions, specialized mechanisms are in place to rapidly degrade Arc mRNA and protein such that they have half-lives of less than 1 h. In addition, we find that while mTOR signaling is regulated by network activity on a similar time scale, mTOR-dependent translational control is not a major regulator of Arc production or degradation suggesting that the signaling pathways underlying homeostatic plasticity are distinct from those mediating synapse-specific forms of synaptic depression.

## Introduction

Homeostatic control mechanisms are prevalent in biological systems and are vital for maintaining physiological parameters within a preferred range (Davis, 2006). Homeostasis is particularly important in the nervous system as neuronal activity must be tightly regulated to preserve stable information flow in neural networks. Multiple mechanisms are in place to maintain network homeostasis and prevent periods of chronically high activity from leading to runaway excitation and periods of low activity from resulting in loss of transmission through the network (Marder and Goaillard, 2006). Importantly, mechanisms that regulate the strength of synapses as a means to normalize excitatory drive must still preserve differences in the weight of individual synapses to allow specific connections to be strengthened or weakened (Turrigiano, 2012).

One well-characterized form of homeostatic plasticity is the scaling of excitatory synapses in response to chronic changes in network activity, which produces a proportional change in strength across the majority of synapses onto a neuron (Turrigiano et al., 1998). This synaptic scaling occurs bi-directionally to counterbalance the effects of altered network activity and is thus, a form of negative feedback. Scaling-down of excitatory synapses in response to chronic increases in network activity requires the activity-regulated protein Arc (Shepherd et al., 2006). In response to prolonged heightened levels of network activity, Arc levels increase and, via interactions with dynamin and endophillin, Arc stimulates the clathrin-dependent endocytosis of synaptic glutamate receptors (Rial Verde et al., 2006; Shepherd et al., 2006; Waung et al., 2008). During periods of low activity, Arc levels drop, which allows the accumulation of synaptic glutamate receptors. This up- or down-regulation of glutamate receptors produces compensatory changes in excitatory drive that are thought to help restore firing rates to their preferred levels. Consistent with a negative feedback function for Arc in regulating neural activity, global deletion of Arc in mice leads to network hyperexcitability and seizures (Peebles et al., 2010).

Arc additionally functions to weaken synaptic strength following activation of metabotropic glutamate receptors (mGluRs), mediating a type of plasticity termed mGluR-dependent long-term depression (mGluR-LTD) (Luscher and Huber, 2010). In this form of synapse-specific plasticity, preexisting Arc mRNA is rapidly and locally translated near activated synapses (Park et al., 2008; Waung et al., 2008). The local accumulation of Arc triggers endocytosis of glutamate receptors at the stimulated synapse, resulting in a long-lasting depression of synaptic responses. Notably, mGluR-LTD requires new protein synthesis through coordinated activation of the mTOR and ERK1/2 signaling pathways (Gallagher et al., 2004; Hou and Klann, 2004; Banko et al., 2006) and is altered in mouse models of autism spectrum disorders which have mutations in molecules involved in translational control (Huber et al., 2002; Bateup et al., 2011; Takeuchi et al., 2013).

The molecular events underlying mGluR-LTD have been well studied, however, less is known about the dynamics of the signaling pathways regulating Arc in response to chronic changes in network activity, a pathway that must be tightly controlled in order to maintain networks within their optimal operating range. For example, it is unknown whether the regulation of Arc in response to changes in network activity also requires signaling through mGluRs, ERK1/2, or mTOR and whether these molecules function within a common pathway. Moreover, while local protein translation is important for mGluR-LTD, Arc is an immediate early gene which is transcriptionally induced in response to activity (Link et al., 1995; Lyford et al., 1995). The relative contribution of translational and transcriptional control to the regulation of Arc during homeostatic plasticity is not well understood.

Here we examine these questions in active networks of cultured mouse hippocampal neurons. We find that changes in Arc levels in response to manipulations of activity are mediated at the level of gene transcription and are independent of mGluRs and translational control through mTOR. Instead, the rapid, bidirectional control of Arc results from ERK1/2-dependent transcription and specialized mechanisms that rapidly degrade Arc mRNA and protein following activity- or transcriptional-blockade. Our findings delineate the temporal dynamics and signaling components of the Arc-dependent negative feedback pathway important for regulating excitatory synaptic drive and neural network activity. Notably, we find that the signaling pathways underlying this form of homeostatic plasticity are largely distinct from those mediating synapse-specific forms of synaptic depression.

## Results

### Basal state kinetics of Arc production and degradation

**Figure 1:**
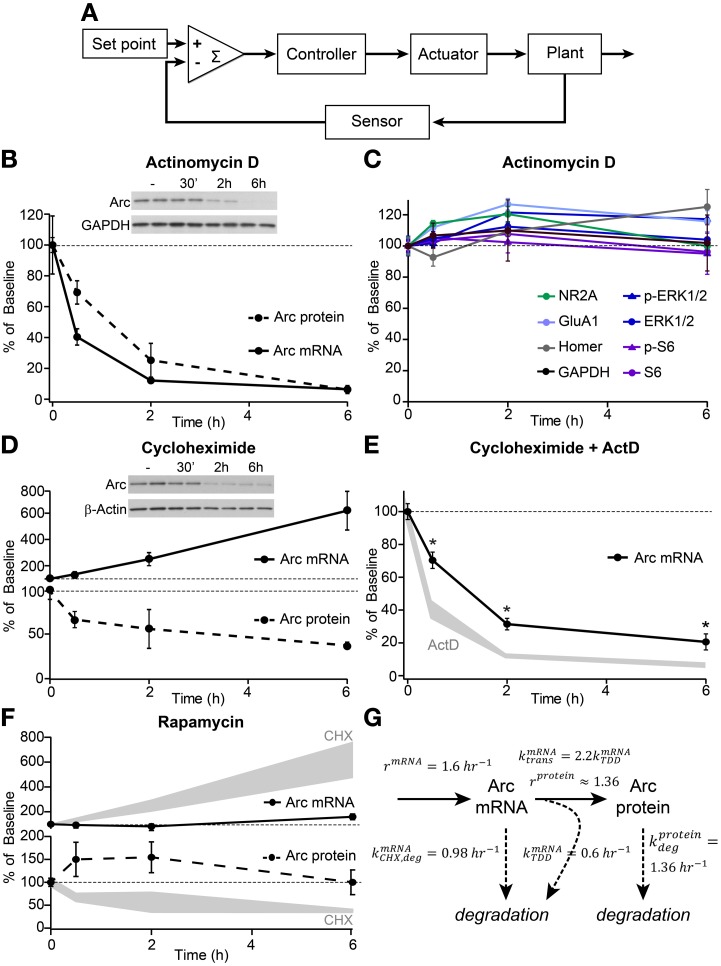
**Arc mRNA and protein exhibit high basal turnover rates and translation-dependent mRNA decay**. **(A)** Schematic depicting the elements of a homeostatic negative feedback system. **(B)** Decay curves of Arc mRNA (measured by quantitative RT-PCR, solid line) and protein (determined by western blotting, dashed line) from dissociated hippocampal cultures following treatment with 8 μM actinomycin D (ActD) for the indicated times in hours. **(C)** Western blotting of the synaptic and signaling proteins indicated in the legend revealed no effects of 6 h treatment with actinomycin D on total protein levels or phosphorylation state. **(D)** Time courses of Arc mRNA accumulation (solid line) and protein degradation (dashed line) following treatment with 10 μM cycloheximide (CHX). **(E)** Decay curves of Arc mRNA levels following combined treatment with 8 μM ActD and 10 μM CHX (black line) versus ActD alone (shaded gray, replotted from **B**). ^*^Indicates significant difference (*p* < 0.05) from ActD alone. **(F)** Treatment with 50 nM rapamycin had no significant effect on Arc mRNA (top panel, solid line) or Arc protein (bottom panel, dashed line) levels. For comparison, Arc mRNA and protein levels following CHX treatment are replotted from **(D)** in shaded gray. **(G)** Diagram detailing experimentally-derived time constants for the production and degradation of Arc mRNA and protein. See methods for the derivation of these values. All data are from 2–3 independent experiments and are represented as mean ± SEM. Dotted lines at 100% indicate baseline levels.

Homeostatic control over a physiological variable can be considered using control system theory (Figure 1A). A negative feedback system typically comprises a comparator that calculates an error signal representing the difference between the operating and desired set points, a controller that amplifies or conditions the error signal, an actuator that alters the properties of the system, and a plant that produces the output of the system. A sensor monitors the output of the plant and provides a feedback signal to the comparator. The stability and robustness of the system depends crucially on the kinetics of the elements such that slow sensors or actuators often lead to oscillations. Conversely, although rapid feedback is often desired to stabilize control systems, overly rapid feedback would prevent dynamic modulation of the system in response to changing external factors.

Using this framework, Arc can be thought of as an actuator of the negative feedback pathway and thus, may determine the temporal scale over which the homeostatic response operates. Therefore, we examined the dynamics of Arc production and degradation in dissociated hippocampal neuronal cultures under basal conditions (Figures 1B–G). Previous studies indicate that Arc levels are tightly controlled by both transcriptional and translational mechanisms (Bramham et al., 2010). We find that under basal conditions, Arc mRNA, measured by quantitative PCR, is acutely sensitive to ongoing transcription such that its levels rapidly decline following transcriptional blockade with actinomycin D (ActD) with an mRNA half-life of ~45 min (Figures 1B,G). Similarly, protein levels decline quickly in ActD indicating a protein half-life of ~90 min (Figures 1B,G). This is an underestimate of the true degradation rate of Arc protein given the ongoing translation from pre-existing, albeit decaying, mRNA. Correcting for the mRNA degradation rate yields an estimate of a protein half-life of ~50 min (see Methods, for discussion of the quantitative model), consistent with a previous report (Rao et al., 2006). The rapid decline of Arc protein is markedly different from the stability of other synaptic and signaling proteins over 6 h of transcriptional blockade (Figure 1C). These data indicate that ongoing transcription is necessary to maintain basal Arc mRNA and protein levels and that processes are in place to rapidly degrade Arc mRNA and protein such that they have half-lives of less than 1 h.

A previous report suggested that Arc mRNA was subject to translation-dependent mRNA decay (TDD) and that this might be a mechanism to tightly control the temporal and spatial activation of Arc (Giorgi et al., 2007). In line with this, globally blocking protein translation with cycloheximide (CHX) resulted in a rapid and robust accumulation of Arc mRNA, whereas Arc protein was degraded in a time-dependent manner (Figure 1D). Additionally, co-application of CHX and ActD slowed the degradation of Arc mRNA relative to application of ActD alone (Figure 1E) providing further evidence that Arc mRNA is degraded in a translation-dependent manner. Notably, the TDD of Arc mRNA was only observed by directly blocking translation with CHX, a drug that inhibits elongation of the polypeptide chain. When we used rapamycin to block mTOR complex 1, which regulates translation initiation factors to control the synthesis of certain classes of mRNAs (Ma and Blenis, 2009), we did not observe significant effects on the global levels of Arc mRNA or protein (Figure 1F). This implies that mTOR is not a primary regulator of Arc protein translation or TDD of Arc mRNA under basal conditions.

The above data can be used to extract rates of Arc mRNA and protein production and degradation in the basal state (Figure 1G), which indicate that Arc mRNA and protein are rapidly turned over and that protein production and mRNA degradation are linked. The rapid rates of production and degradation demonstrate that essentially all of the Arc mRNA and protein are replaced every 1–2 h.

### Dynamics of Arc regulation by neural network activity

In the context of a negative feedback system, the fast kinetics and high basal turnover rate of Arc described above should allow its rapid and dynamic regulation in response to differences between the desired and actual levels of neural activity. In order to better understand the dynamic regulation of Arc following changes in network activity, we either eliminated activity by blocking action potentials with the voltage-gated sodium channel antagonist TTX or increased network activity by blocking inhibitory neurotransmission with the GABA receptor antagonist picrotoxin.

**Figure 2:**
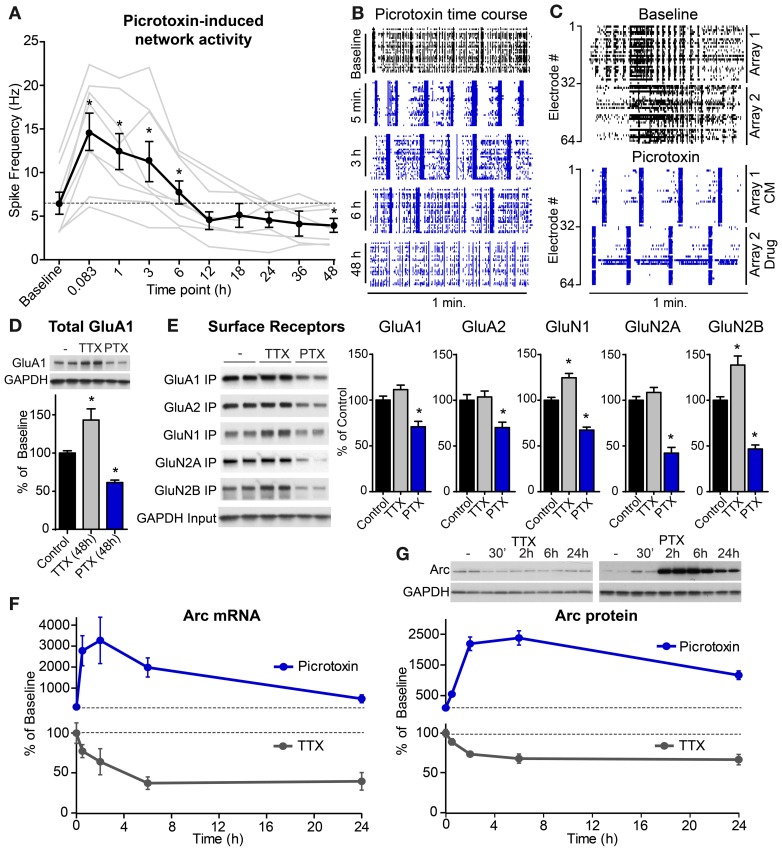
**High levels of network activity induce Arc mRNA and protein, down-scaling of glutamate receptors, and homeostatic plasticity of network spiking rates**. **(A)** Average spike rate per electrode (*n* = 8 experiments, plotted individually in gray) from hippocampal neurons plated onto multi-electrode arrays and treated with 0.5 μM picrotoxin for the indicated durations in hours. **(B)** Raster plots of multi-unit activity recorded at different times following picrotoxin treatment. Each line represents a single spike detected in a given channel during a one minute recording. **(C)** Neurons were plated onto two sides of a split-chamber MEA and baseline activity was recorded from both cultures at DIV 14 (top panel). The neurons on electrodes #1–32 (Array 1) were then treated with conditioned media (CM) from another culture previously incubated with 1 μM picrotoxin for six hours. The neurons on electrodes #33–64 (Array 2) were treated with 1 μM freshly prepared picrotoxin (Drug). Spiking responses were recorded five minutes later (bottom panel). **(D)** Total levels of GluA1 protein normalized to GAPDH loading control following 48 h treatment with TTX or picrotoxin (PTX). **(E)** Left: western blots from a biotin surface-protein labeling experiment from cultures treated for 48 h with TTX or PTX. Surface proteins were labeled with biotin and immunoprecipitated (“IP”). “Input” panel shows equivalent GAPDH in total cell lysates from each condition. Right: quantification of surface levels of glutamate receptor subunits following 48 h treatment with TTX or PTX (*n* = 6 samples). ^*^Indicates significant difference (*p* < 0.05) from baseline. **(F)** Time courses of Arc mRNA levels following treatment with 50 μM picrotoxin (blue line) or 1 μM TTX (gray line) for the indicated times in hours. **(G)** Top: representative western blots of Arc protein levels following treatment with TTX (left) or picrotoxin (PTX, right). Arc values were normalized to GAPDH loading control for each sample. Bottom: average Arc protein levels for neurons treated with picrotoxin (blue) or TTX (gray). All data are represented as mean ± SEM. Dotted lines at 100% indicate baseline levels.

We first examined the temporal dynamics of these pharmacological manipulations on network activity by monitoring spiking responses in real time from neurons plated onto multi-electrode arrays (MEAs). We applied TTX to cultures at 21 DIV and found that within 5 min TTX blocked all action potential firing (baseline spike frequency = 7.52 Hz; TTX = 0 Hz). This suppression of activity was maintained for at least 48 h in the continued presence of TTX. Conversely, picrotoxin robustly increased the frequency of action potentials and converted network activity to a bursting pattern within 5 min of application (Figures 2A–C). Spiking rates recovered to baseline levels after 12 h of picrotoxin treatment and dropped slightly below the baseline by 48 h (Figures 2A,B), suggestive of homeostatic adjustment of network activity levels. These activity dynamics likely reflect engagement of activity-dependent homeostatic processes rather than loss of effectiveness or degradation of the drug as picrotoxin-conditioned media stimulated network activity and bursting to a similar degree as fresh picrotoxin in a pair of cultures plated on a split chamber MEA (Figure 2C).

Multiple homeostatic systems are likely operating in parallel to restore network activity levels following blockade of inhibition. Since action potentials in hippocampal projection neurons are largely driven by excitatory synaptic inputs, one important homeostatic mechanism is down-regulation of excitatory drive by removing glutamate receptors from synapses. We found evidence for this at the biochemical level as total protein levels of the AMPA receptor subunit GluA1 were decreased 48 h after picrotoxin treatment (Figure 2D). Conversely, activity blockade, with TTX increased total levels of GluA1 protein (Figure 2D). However, in this case, altering glutamatergic synaptic strength cannot restore activity as TTX blocks spiking downstream of synaptic drive. Notably, we found that many glutamate receptor subunits showed some degree of homeostatic compensation at the level of receptor surface expression (Figure 2E). This suggests that in this paradigm, excitatory drive is modulated non-selectively, either by adjusting levels of both AMPA- and NMDA-type glutamate receptors (Watt et al., 2000), or by changing the overall number of excitatory synapses (Goold and Nicoll, 2010).

Arc-mediated endocytosis of surface glutamate receptors (Chowdhury et al., 2006; Rial Verde et al., 2006) is a key part of the homeostatic response to elevated activity and its down-regulation in low activity conditions is necessary to increase synaptic glutamate receptors (Shepherd et al., 2006). Therefore, as an actuator of this negative feedback pathway we investigated the temporal dynamics of Arc regulation by activity as well as the upstream pathways controlling its induction. Through this analysis we also determined whether the processes that tightly control Arc production and degradation in the basal state can account for the kinetics of Arc modulation by activity. We found that activity blockade with TTX significantly reduced Arc mRNA levels within 2 h to about 64% of basal levels (Figure 2F). Compared to complete transcriptional blockade (see Figure 1B), TTX treatment resulted in a ~3 fold slower rate of mRNA decay, indicating that passive degradation of Arc mRNA was sufficient to account for its loss following activity blockade. In parallel, activity blockade resulted in a rapid drop in Arc protein levels (Figure 2G). Arc protein re-equilibrated at a similar or moderately slower rate to that of Arc mRNA following TTX and Arc protein in the presence of ActD, indicating that the high passive turnover of Arc protein is sufficient to explain its rate of decay in TTX.

In contrast to activity blockade, elevated network activity caused a rapid and robust increase in Arc mRNA within 30 min, peaking with 2 h of picrotoxin treatment (Figure 2F). Similarly, Arc protein increased with a delay relative to the mRNA, peaking 2–6 h after picrotoxin application (Figure 2G). The time when Arc protein levels were maximally high corresponded to the time when there was the steepest drop in network activity (see Figure 2A) despite the continued presence of picrotoxin. Therefore, Arc may have been acting to normalize activity over this time scale. Taken together, these data indicate that Arc levels are rapidly and robustly modulated in a bidirectional manner within a few hours of altered network activity, a time course consistent with subsequent restoration of network activity.

### Activity sensors and control pathways regulating the production of Arc

**Figure 3:**
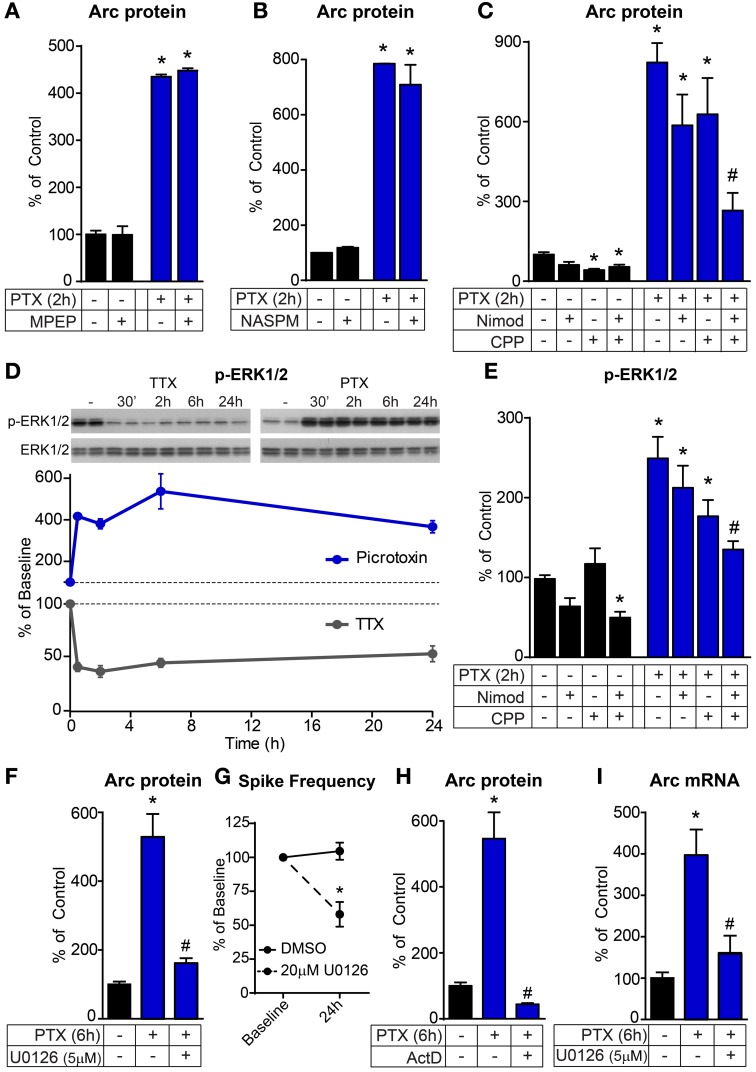
**The activity-dependent induction of Arc requires ERK1/2-dependent transcription**. **(A–C)** Bar graphs displaying western blot data of Arc protein normalized to GAPDH loading control. Hippocampal cultures were treated for 2 h with 50 μM picrotoxin (PTX) and pre-incubated for 30 min with either 4 μM MPEP **(A)**, 10 μM NASPM **(B)**, 5 μM nimodipine (Nimod) and/or 10 μM CPP **(C)**. **(D)** Top: representative western blots of phosphorylated ERK1/2 (p-ERK1/2, Thr202/Tyr204) normalized to total ERK1/2 protein following treatment with TTX (left) or picrotoxin (PTX, right) for the indicated times in hours. Bottom: average p-ERK1/2 levels in neurons treated with picrotoxin (blue) or TTX (gray) for the indicated times. Dotted lines at 100% indicate baseline levels. **(E)** Western blot data of p-ERK1/2 normalized to total ERK1/2 following 2 hour treatment with nimodipine, CPP, and/or picrotoxin as indicated. **(F,H)** Western blot data of Arc protein normalized to GAPDH following six hour treatment with picrotoxin with and without 5 μM U0126 **(F)** or 8 μM actinomycin D (ActD, **H**). **(G)** Spike frequency expressed as a percentage of baseline determined by multi-electrode array recordings of pairs of cultures treated with DMSO or 20 μM U0126 for 24 h. **(I)** Quantitative RT-PCR data of Arc mRNA levels following six hour treatment with picrotoxin with and without 5μM U0126. All data are from 2–3 independent experiments and are represented as mean ± SEM. ^*^Indicates significant difference (*p* < 0.05) from control baseline level. ^#^ Indicates significant difference (*p* < 0.05) from picrotoxin treated.

After establishing the temporal dynamics over which the Arc homeostatic pathway operates, we examined the upstream signals that might serve as homeostatic sensors linking changes in network activity to Arc production. During mGluR-LTD, activation of group 1 mGluRs leads to the local dendritic synthesis of Arc protein which removes AMPA receptors from the stimulated synapse resulting in long-term depression (Park et al., 2008; Waung et al., 2008). To determine whether mGluRs mediate the network activity-dependent production of Arc we blocked group 1 mGluRs during picrotoxin treatment. We found no effect of either the selective mGluR5 antagonist MPEP (Figure 3A) or the mGluR1/5 antagonist AIDA (Arc protein in picrotoxin = 1173 ± 89.9% of control; Picrotoxin + AIDA = 1131 ± 61.3% of control) on the induction of Arc protein by activity indicating that different mechanisms regulate the production of Arc during global network activity changes and synapse-specific mGluR-LTD.

Calcium is an important second messenger in neurons that is vital for transducing network activity into long-term changes in neuronal function in a variety of plasticity paradigms. However, there are many possible routes for calcium entry and differential engagement of calcium sources has distinct consequences on the neuronal response to activity (Greer and Greenberg, 2008). Therefore, we tested the contribution of several calcium sources to the activity-dependent production of Arc. Although they are required for the expression of several forms of synaptic plasticity including synaptic scaling (Perkinton et al., 1999; Tian and Feig, 2006; Beique et al., 2011), we found that calcium permeable AMPA receptors were not required for the induction of Arc protein by activity (Figure 3B). In contrast, NMDA receptors (NMDAR) and L-type voltage-gated calcium channels (L-VGCC) each made a small contribution and together accounted for most of the activity-dependent upregulation of Arc protein by picrotoxin (Figure 3C). Notably, blockade of either NMDA receptors alone or both NMDARs and L-VGCCs reduced basal levels of Arc protein suggesting that ongoing calcium entry through these channels in response to normal activity maintains basal levels of Arc (Figure 3C). Thus, NMDARs and L-VGCCs are key components of the activity sensor upstream of Arc.

One calcium-responsive pathway that is vital for a number of synaptic processes and forms of plasticity, and therefore, may be an integral part of the homeostatic controller, is the MEK-ERK pathway. Consistent with this possibility, we found that ERK1/2 phosphorylation was rapidly, robustly, and bi-directionally modulated by network activity (Figure 3D). The phosphorylation of ERK1/2 occurred on a time scale preceding the changes in Arc mRNA and protein (see Figures 2F,G) and could therefore, lie downstream of NMDARs and L-VGCCs to regulate synthesis of Arc. In line with this, combined blockade of NMDARs and L-VGCCs significantly reduced both the basal and activity-induced phosphorylation of ERK1/2 (Figure 3E). To determine whether MEK-ERK signaling was directly responsible for the upregulation of Arc by activity, we blocked the pathway with U0126 and found that it prevented the induction of Arc protein by picrotoxin (Figure 3F). We used U0126 at a concentration of 5 μ M because we found that higher doses acutely affected network activity (20 μ M reduced activity by 66% within 5 min) resulting in a chronic reduction in spiking activity (Figure 3G). This suggests that high concentrations of U0126 may have off-target effects that directly affect neuronal activity (Yuan et al., 2006).

The MEK-ERK pathway can regulate both transcription and translation in neurons via activation of different downstream targets (Kelleher et al., 2004; Wiegert and Bading, 2011). In this paradigm we found that the induction of Arc protein by picrotoxin was abolished by the transcriptional inhibitor ActD (Figure 3H) indicating that the regulated step is at the level of transcription. Therefore, the synthesis of new Arc mRNA is required and acute translation of preexisting mRNA is not a major contributor to the induction of Arc protein by global network activity. We find that ERK1/2 is the primary regulator of activity-induced Arc transcription because the up-regulation of Arc mRNA with picrotoxin was significantly reduced by pre-incubation with U0126 (Figure 3I).

**Figure 4:**
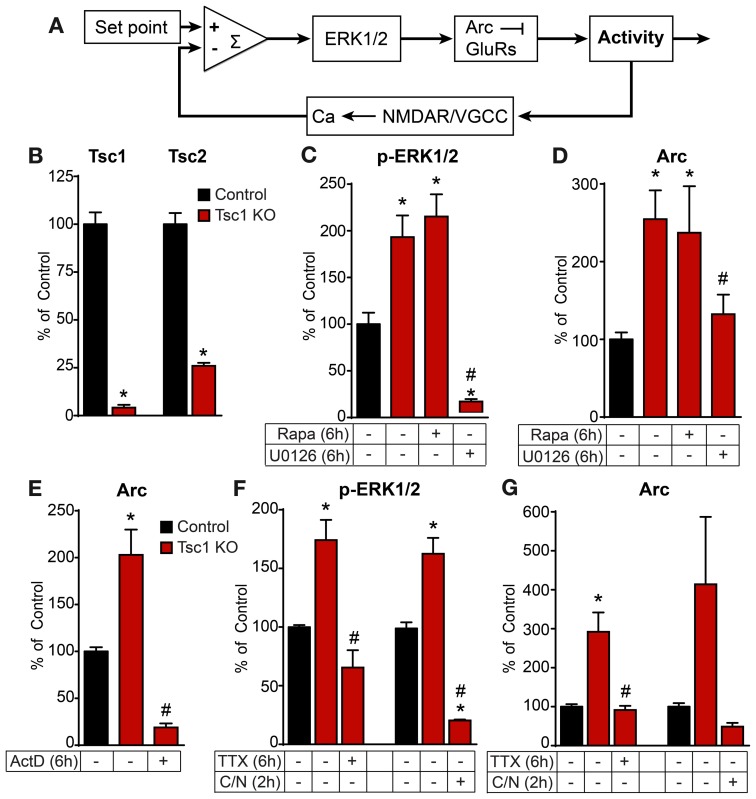
**The ERK1/2-Arc negative feedback pathway is tonically active in Tsc1 KO neurons and is independent of mTOR**. **(A)** Schematic depicting the ERK1/2-Arc negative feedback pathway regulating hippocampal network activity. **(B)** Bar graphs show western blot data of Tsc1 and Tsc2 protein levels normalized to GAPDH or β-Actin loading control on DIV 14 from control (black) and Tsc1 KO (red) cultures. **(C,D)** Western blot data of p-ERK1/2 normalized to total ERK1/2 **(C)** and Arc protein normalized to β-Actin loading control **(D)** in control and Tsc1 KO cultures following six hour treatment with 50 nM rapamycin (Rapa) or 5 μM U0126. **(E)** Western blot data of Arc protein normalized to β-Actin loading control in control and Tsc1 KO cultures treated for six hours with 8 μM actinomycin D (ActD). **(F,G)** Western blot data of p-ERK1/2 **(F)** and Arc **(G)** in control and Tsc1 KO neurons with and without 1 μM TTX (six hours) or 10 μM CPP plus 5 μM Nimodipine (C/N, two hours). All data are from 2–3 independent experiments and are represented as mean ± SEM. ^*^Indicates significant difference (*p* < 0.05) from control. ^#^Indicates significant difference (*p* < 0.05) from untreated Tsc1 KO.

Taken together, these results support the following model for the negative feedback regulation of network activity in hippocampal cultures (Figure 4A). Chronic, high levels of network activity result in calcium influx through NMDARs and L-VGCCs which activate the MEK-ERK pathway that is largely responsible for activating the transcription of Arc mRNA. Arc mRNA is made into protein, causing subsequent degradation of the mRNA, which then regulates surface glutamate receptor levels as a means to normalize excitatory drive. We have observed the bidirectional, temporal dynamics of the components of this feedback loop in wild-type cells where this pathway, and presumably others, are capable of normalizing network activity in response to a global perturbation. However, what happens to this feedback pathway in a pathological state in which activity is constitutively high?

We have previously shown that conditional deletion of the mTOR negative regulator, Tsc1, results in chronically high hippocampal network activity levels *in vitro* and severe seizures *in vivo* (Bateup et al., 2013). In that study we observed tonic engagement of homeostatic scaling of glutamate receptors in an attempt to counteract the high levels of activity. Since the MEK-ERK and mTOR pathways converge at the biochemical level (Mendoza et al., 2011) and work in concert to regulate several types of synaptic plasticity (Banko et al., 2006; Gelinas et al., 2007; Costa-Mattioli et al., 2009), we further investigated ERK1/2 signaling in Tsc1 KO neurons and the interactions between mTOR and ERK1/2 during homeostatic plasticity. To do this we prepared dissociated hippocampal cultures from mice with conditional alleles of *Tsc1* (Kwiatkowski et al., 2002) and added high titer lentivirus expressing either synapsin-driven GFP (Control) or GFP-IRES-Cre (Tsc1 KO) to delete *Tsc1* and cause up-regulation of mTOR signaling. By 14 DIV we observed near complete loss of Tsc1 protein and destabilization and degradation of its binding partner Tsc2 (Figure 4B).

Similar to the high levels of Arc we previously observed in Tsc1 KO neurons (Bateup et al., 2013, see also Figure 4D), we found that phosphorylated ERK1/2 levels were constitutively high in an mTOR-independent manner (Figure 4C). Furthermore, activation of the ERK-dependent transcriptional pathway was directly responsible for the high levels of Arc following loss of Tsc1 as blocking MEK-ERK signaling with U0126 (Figure 4D) or blocking transcription with ActD (Figure 4E) reduced the tonic upregulation of Arc in Tsc1 KO neurons. Additionally, blocking either the activity-drive with TTX or the activity sensors NMDAR/L-VGCCs was sufficient to reverse the high levels of both p-ERK (Figure 4F) and Arc (Figure 4G) in Tsc1 KO cultures. These data support the model described in Figure 4A and demonstrate that the entire activity-dependent negative feedback module is tonically active in Tsc1 KO networks *in vitro*. This pathway is presumably chronically engaged because down-scaling of excitatory drive is not able to compensate for the primary loss of inhibition in the network caused by loss of Tsc1 (Bateup et al., 2013).

**Figure 5:**
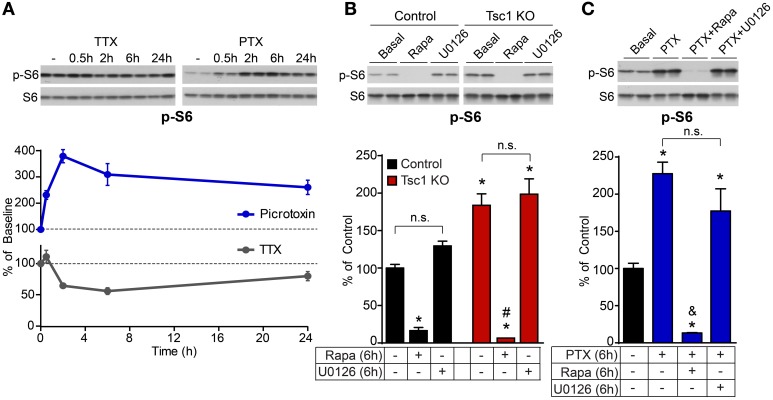
**ERK1/2 blockade does not impact basal or activity-regulated phosphorylation of the mTOR pathway target S6**. **(A)** Western blot data of average phosphorylated S6 (p-S6, Ser240/244) levels normalized to total S6 in control neurons treated with picrotoxin (PTX, blue) or TTX (gray) for the indicated times. Dotted lines at 100% indicate baseline levels. **(B)** Western blot data of phosphorylated S6 in control (black) and Tsc1 KO neurons (red) following six hour treatment with 50 nM rapamycin (Rapa) or 5 μM U0126. **(C)** Western blot data of phosphorylated S6 in control neurons following six hour treatment with 50 μM picrotoxin (PTX), with or without 50 nM rapamycin or 5 μM U0126. All data are from 2–3 independent experiments and are represented as mean ± SEM. ^*^Indicates significant difference (*p* < 0.05) from untreated control. ^#^Indicates significant difference (*p* < 0.05) from untreated Tsc1 KO. ^&^Indicates significant difference (*p* < 0.05) from picrotoxin treated control. n.s. indicates no significant difference between groups.

We also found that mTOR signaling was bi-directionally regulated by network activity in control neurons on a time scale similar to Arc and p-ERK1/2 (Figure 5A). However, this was independent of ERK1/2 as blocking MEK-ERK signaling did not significantly affect phosphorylation of the mTOR target S6 in control or Tsc1 KO neurons (Figure 5B), nor did it affect the activity-dependent up-regulation of p-S6 (Figure 5C). This is in contrast to the complete loss of phosphorylated S6 following treatment with the mTOR inhibitor rapamycin (Figures 5B,C), demonstrating that mTOR is the primary signaling pathway regulating p-S6 at Ser240/244 under basal and high activity conditions. Thus, together with our previous findings showing that mTOR is a positive regulator of network activity by reducing levels of inhibition (Bateup et al., 2013), we conclude that ERK1/2 and mTOR comprise independent and opposing feedback pathways that control hippocampal network excitability via distinct mechanisms.

## Discussion

In this study, we investigated the dynamics of a homeostatic signaling pathway induced by network activity in hippocampal neurons. Diverse molecules and mechanisms have been identified that control homeostatic plasticity in neurons, highlighting the importance of these processes for network stability (Pozo and Goda, 2010). Here we investigated the kinetics and regulation of Arc, which is a key post-synaptic homeostatic actuator responsible for scaling synaptic glutamate receptors following chronic activity manipulations (Shepherd et al., 2006). Specifically, we determined the contribution of transcriptional and translational control to the basal and activity-dependent induction of Arc, the relevant signaling pathways responsible for regulating Arc, and the time course of these signaling changes as they relate to the restoration of network activity. We find that an ERK1/2-dependent transcriptional pathway active within 1–2 h of up-regulated network activity is responsible for regulating Arc and that mechanisms are in place to rapidly degrade Arc mRNA and protein when activity is low. In addition we find that although mTOR signaling is also regulated by network activity, mTOR-dependent translational control is not a major regulator of global Arc levels during homeostatic plasticity.

### Kinetics of Arc production and degradation

In this study we provide quantitative analysis of the kinetics of production and degradation of Arc mRNA and protein under basal and activity-modulated conditions. We find that ongoing transcription is necessary to maintain basal Arc mRNA and protein levels and that specialized processes are in place to rapidly degrade Arc mRNA and protein such that they have half-lives of less than 1 h. One such mechanism is translation-dependent decay of Arc mRNA (Giorgi et al., 2007), which is revealed by our finding that blockade of protein translation caused accumulation of Arc mRNA and slowed its degradation rate when transcription was blocked. Other mechanisms contributing to the tight temporal and spatial control of Arc include specialized machinery for rapid, activity-induced transcription by stalled Pol II (Saha et al., 2011) and protein degradation via the activity-regulated ubiquitin ligase Ube3A (Greer et al., 2010). Interestingly, despite the range of dynamic regulatory mechanisms for Arc, we find that mTOR and mTOR-dependent translation are not important for controlling Arc production or degradation under basal conditions or following chronic changes in network activity in hippocampal cultures. These results are in close agreement with a recent study examining the dynamics of Arc in human neuroblastoma cells in response to muscarinic receptor stimulation (Soule et al., 2012).

### Activity-dependent regulation of Arc

We investigated the temporal dynamics of the signaling pathways regulating Arc in response to changes in network activity and determined the time frame over which this homeostatic pathway acts. We find that blocking inhibition with picrotoxin results in a rapid (within 5 min) increase in spiking frequency, and a conversion of network activity to a highly correlated, bursting pattern. We monitored network activity in the continued presence of picrotoxin and found a pattern indicative of homeostatic restoration of spiking rate by 12 h. We observed compensatory changes in both AMPA- and NMDA-type glutamate receptors at the biochemical level consistent with the possibility that changes in excitatory drive were acting to normalize network spike rates. Since we found changes in both surface and total levels of glutamate receptors, one possibility is that Arc stimulates removal of receptors from synapses which leads to subsequent degradation (Fu et al., 2011) or full-scale loss of excitatory synapses via other mechanisms.

The regulation of Arc has been studied in several contexts with differential contribution of translational and transcriptional control (Bramham et al., 2010; Shepherd and Bear, 2011). Here we have investigated the regulation of Arc by chronic (several hours) changes in network activity in dissociated hippocampal cultures. We find that the rate-limiting step is mRNA transcription which is dependent on the activation of ERK1/2. Activity-responsive elements have been identified in the Arc promoter that contain binding sites for SRF, CREB, and MEF2 (Kawashima et al., 2009; Pintchovski et al., 2009). ERK1/2 could impact the activity of these elements directly via phosphorylation of CREB, or indirectly through intermediate kinases and transcription factors such as MSK1/2, RSK, and Elk-1. An ERK1/2-dependent transcriptional pathway also regulates Arc during the induction of LTP following high frequency stimulation or BDNF infusion into the dentate gyrus *in vivo* (Ying et al., 2002; Panja et al., 2009) and in cortical cultures *in vitro* (Rao et al., 2006). It is important to note, however, that these studies used U0126 at concentrations of 20 μM and higher which may have directly blocked network activity (see Figure 3G), thereby eliminating the signal necessary for activity-dependent Arc upregulation.

### Mechanisms of synapse-specific vs. homeostatic plasticity

Although some signaling components might be shared, we find that the mechanisms involved in homeostatic plasticity are largely distinct from those observed in synapse-specific plasticity. For example, in addition to synaptic scaling (Shepherd et al., 2006), Arc is also required for mGluR-LTD (Park et al., 2008; Waung et al., 2008) and BDNF-induced long-term potentiation (Messaoudi et al., 2007). In mGluR-LTD, activation of mGluRs results in local synthesis of pre-existing Arc mRNA which requires both ERK1/2 and mTOR-dependent translational control (Gallagher et al., 2004; Hou and Klann, 2004; Banko et al., 2006; Antion et al., 2008). Similarly, in response to application of the neurotrophin BDNF, the translation of Arc protein is increased in an mTOR and ERK1/2-dependent manner (Takei et al., 2004; Briz et al., 2013). In contrast to these forms of plasticity, which occur relatively rapidly and locally affect a sub-set of synapses, we find that during homeostatic plasticity Arc is induced by an ERK1/2-dependent transcriptional pathway which is independent of translational control by mTOR. Together these studies suggest that in response to different types of stimuli, Arc can be induced via distinct and potentially non-overlapping mechanisms. Notably, ERK1/2-dependent but mTOR-independent regulation of Arc has also been reported *in vivo* following high frequency stimulation that evokes long-term potentiation in the dentate gyrus (Panja et al., 2009). Although the signaling mechanisms in that study parallel what we find here, it is important to note that LTP and synaptic scaling produce opposite effects on synaptic strength. Key differences between LTP and homeostatic plasticity are the spatial and temporal scales over which they operate. It is possible that Arc acts on different substrates or interacts with distinct partners to mediate these contrasting types of plasticity. In support of this, it was recently shown that in response to prolonged (>8 h) high levels of activity, Arc translocates to the nucleus where it down-regulates the expression of GluA1 resulting in a global decrease in synaptic strength. By contrast, at earlier time points after the activity stimulation, Arc was excluded from the nucleus and it is possible that during this time window, Arc might interact with synaptic proteins in the cytoplasm to locally facilitate LTP via other mechanisms (Korb et al., 2013).

In addition to studying the signaling mechanisms involved in the production of Arc, we also investigated the relationship between the mTOR and ERK1/2 signaling pathways during homeostatic plasticity. Studies in non-neuronal cells have demonstrated that ERK1/2 activates mTOR signaling via inhibition of Tsc1/2 (Ma et al., 2005), suggesting that ERK1/2 and mTOR may operate in a common pathway. Indeed, as discussed above, mTOR and ERK1/2 appear to both be required for certain forms of LTD and LTP. Here we find that ERK1/2 and mTOR signaling are largely independent as up- or down-regulation of mTOR had no effect on the activity-dependent induction of ERK1/2 and blockade of ERK1/2 signaling did not perturb basal or activity-regulated mTOR signaling. This may reflect cell-type specific differences in signaling mechanisms between proliferating cells and differentiated neurons (Ma and Blenis, 2009). Alternatively, homeostatic plasticity and synapse-specific plasticity operate on different temporal and spatial scales. Here we have investigated global signaling responses to chronic manipulations of network activity over several hours to days. Our findings do not preclude the possibility that ERK1/2 and mTOR work together in other plasticity paradigms operating on subclasses of targets in more spatially restricted domains.

Taken together, our findings define the temporal dynamics and functional relationships between mTOR, ERK1/2 and Arc during homeostatic plasticity in mouse hippocampal neurons. Since disruptions in synaptic and network homeostasis may be a common pathophysiology in neurodevelopmental disorders (Toro et al., 2010; Zoghbi and Bear, 2012), our findings elucidate possible points of vulnerability and intervention that might be relevant for disease.

## Materials and methods

All animal handling was performed in accordance with guidelines approved by the Harvard Institutional Animal Care and Use Committee and federal guidelines.

### Drugs and antibodies

Reagents were obtained from the following sources: U0126 (Calbiochem); Rapamycin, Actinomycin D, and NASPM (Sigma); Picrotoxin, TTX, MPEP, AIDA, Nimodipine, CPP, and CHX (Tocris). Antibodies for western blotting were obtained from: Arc (Santa Cruz and Synaptic Systems), Tsc1 and Tsc2 (Bethyl Labs), β-Actin (Sigma), GluA1 (Calbiochem), GluA2, GluN1, and GluN2A (all from Millipore), GluN2B (BD Biosciences), Homer (Santa Cruz), p-S6 (S240/244), total S6, p-ERK1/2 (Thr202/Tyr204), total ERK1/2, and GAPDH (all from Cell Signaling).

### Dissociated hippocampal cultures

Primary dissociated hippocampal cultures were prepared from P0-1 C57Bl/6 (Charles River) or *Tsc1*^*fl/fl*^ mice (Kwiatkowski et al., 2002) using standard protocols. Neurons were maintained in Neurobasal media (GIBCO) with glutamine, pen/strep, and B-27 supplement (GIBCO). Cytosine arabinoside (Sigma) was added at 5 DIV to prevent glial proliferation. For biochemical experiments 1.8–2 × 10^5^ cells were plated onto 24-well plates pre-coated with Poly-D-Lysine (PDL). For cultures made from *Tsc1*^*fl/fl*^ mice, lentivirus prepared by the Harvard Gene Therapy Initiative (HGTI) expressing either GFP (6 × 10^8^ IU/mL, Control) or GFP-IRES-Cre (2.1 × 10^8^ IU/mL, Tsc1 KO) from the synapsin promoter was added at 2 days *in vitro* (DIV).

### Western blotting

Unless otherwise indicated, cells were harvested at 14 DIV in lysis buffer containing 2mM EDTA, 2 mM EGTA, 1% Triton-X, 0.5% SDS in PBS with Halt phosphatase inhibitor cocktail (Thermo) and Complete mini EDTA-free protease inhibitor cocktail (Roche). Total protein was determined by BCA assay (Pierce), and 10–15 μg of protein in 4 × Laemmli sample buffer were loaded onto Tris-HCl gels (Bio-Rad). Proteins were transferred to PVDF membranes, blocked in 5% milk in TBS-Tween for 1 h at room temperature (RT), and incubated with primary antibodies overnight at 4°C. Blots were incubated with HRP-conjugated secondary antibodies (Bio-Rad) for 1 h at RT, washed, incubated with chemiluminesence substrate (Perkin-Elmer), and developed on Kodak Bio-Max film. Bands were quantified by densitometry using Image J software. Phospho-proteins were normalized to their respective total proteins and non-phospho-proteins were normalized to either a GAPDH or β-Actin loading control.

### Quantitative RT-PCR

RNA was extracted from neuronal lysates using an RNeasy kit (Qiagen) with on-column DNAse digestion. RNA levels and purity were assessed with a NanoDrop spectrophotometer. Reverse transcription was performed on 180 ng of total RNA using oligo dT primers and Thermoscript reverse transcriptase (Invitrogen) at 50°C. Real-time PCR was performed in an ABI Prism 7000 (Applied Biosystems) with Platinum SYBR green qPCR SuperMIX-UDG with ROX (Invitrogen). Values for Arc were normalized to GAPDH for each sample. Primers for the analysis of endogenous gene expression were:

Arc forward: 5′-ACC CAC TCC CCA AGA CCC T-3′
Arc reverse: 3′-GCA CTT CCA TAC CCC TCT GG-3′
GAPDH forward: 5′-TTC ACC ACC ATG GAG AAG GC-3′
GAPDH reverse: 5′-CCC TTT TGG CTC CAC CCT-3′

### Modeling of Arc mRNA and protein dynamics

The dynamics of Arc mRNA and protein production and degradation in the basal state were modeled using zero and first orders kinetics (Figure 1E). Since the absolute concentrations of mRNA and protein are unknown, rates are expressed relative to the steady state concentration and have units of h^−1^. Therefore, in order to distinguish zero order production rates (i.e., constants) and first order degradation and translation rates (i.e., proportional) we use *r* for the former and *k* for the later. Exponential fit to the time course of mRNA decay in the basal state after addition of ActD indicates fast first-order rate constants of decay *k*^mRNA^_deg_ = 1.6 h^−1^. Similarly, protein levels decline quickly in ActD such that the apparent rate of protein degradation is *k* ^protein^_app,deg_ = 0.8 h^−1^, reflecting the balance of protein production from residual mRNA and the decay of protein due to degradation. Correcting for the mRNA degradation rate, yields an estimate of the protein degradation rate of $k_{\text{deg}}^{\text{protein}}\approx \sqrt{k_{\text{mRNA},\text{deg}}^{2}-k_{\text{protein},\text{app},\text{deg}}^{2}}=1.36{\text{ h}}^{-1}$.

In the basal state, addition of the broad translational inhibitor CHX led to accumulation of Arc mRNA. Co-application of CHX and ActD slowed the degradation of Arc mRNA relative to application of ActD alone to *k*^mRNA^_CHX,deg_ = 0.98 h^−1^ (Figure 1D). This indicates that 40% of the decay of Arc mRNA in the basal state is via a CHX, and presumably translation, sensitive mechanism (*k*^mRNA^_TDD_ = *k*^mRNA^_deg_ − *k*^mRNA^_deg,CHX_ = 0.6 h^−1^).

Two estimates of rates of mRNA production in the basal state can be made based on these data. First, given steady state, the rate of Arc mRNA production and degradation must be balanced. This indicates that Arc mRNA must be produced at *r*^mRNA^ = 1.6 h^−1^ where *r* is used to designate the zero order fixed production rate at steady state in basal conditions and to contrast to the first order *k*. Separately, the initial rate of accumulation of Arc in the presence of CHX is 0.9/h, which when corrected for the rate of translation-independent decay, renders an estimate of *r*^mRNA^ = 1.9 h^−1^, in relatively good agreement with the above independent estimate of 1.6.

### Multi-electrode array recordings

Dissociated hippocampal cultures were prepared as above and plated onto MED64 single or dual-chamber probes (MED-P515A or MED-P5D15A, AutoMate Scientific) pre-coated with PDL and laminin (Invitrogen) at a density of ~4.2 × 10^3^ cells/mm^2^. The plating area was restricted to achieve complete coverage of the central planar electrode array while avoiding plating cells on the outer reference electrodes. Two minute recordings were performed in a 37°C/5% CO_2_ incubator with a MED64 Multi-electrode Array System using a Panasonic 64-channel amplifier and Mobius software (AutoMate Scientific). Spikes were detected using Mobius software with the threshold set at ± 0.009 mV (≥2 fold the baseline). Subsequent analysis was performed in Igor Pro (Wavemetrics) using custom software. Data are presented as average spike rate per electrode, determined by calculating the individual spike rate in Hz for each electrode and averaging this value across all 32 or 64 channels in the array.

### Biotin surface receptor labeling

Dissociated hippocampal cultures were plated onto PDL-coated 12-well plates at a density of 5 × 10^5^ cells per well. At 12 DIV cultures were treated with either vehicle, 1 μM TTX or 50 μM picrotoxin. Forty-eight hours later cells were rinsed briefly in ice-cold 1 × DBPS (+Ca/Mg) and incubated in a 1 mg/mL solution of Sulfo-NHS-SS-Biotin (Thermo) for 30 min at 4°C under non-permeabilizing conditions. Cells were washed 3 × 5 min in cold 100 mM glycine, washed briefly in 1 × DPBS, and harvested in 100 uL RIPA buffer (Sigma) with Halt phosphatase inhibitor cocktail (Thermo) and Complete mini EDTA-free protease inhibitor cocktail (Roche). 25 uL of lysate was removed to determine total protein concentration and retained as the input fraction for western blotting. Equal amounts of protein were added to 25 uL of washed NeutrAvidin UltraLink Resin (Thermo) and rotated overnight at 4°C. Resin was washed 3 times in lysis buffer and Biotin-labeled proteins were eluted by boiling for 5 min in Laemmli sample buffer and analyzed by western blotting as above.

### Statistical analysis

For comparisons between two groups, a two-tailed paired or unpaired student's *t*-test was used. For comparisons between multiple groups including time course data, a One-Way ANOVA with Bonferroni correction for multiple comparisons was used.

## Author contributions

Helen S. Bateup and Bernardo L. Sabatini designed experiments and wrote the manuscript. Helen S. Bateup performed primary data analysis and Bernardo L. Sabatini generated the quantitative model based on experimental data. Cassandra L. Denefrio and Jessica L. Saulnier generated primary neuronal cultures. Cassandra L. Denefrio and Caroline A. Johnson performed western blotting experiments. Helen S. Bateup and Cassandra L. Denefrio performed multi-electrode array recordings and quantitative RT-PCR analysis.

### Conflict of interest statement

The authors declare that the research was conducted in the absence of any commercial or financial relationships that could be construed as a potential conflict of interest.

## References

[B1] Antion M. D. Hou L. Wong H. Hoeffer C. A. Klann E. (2008). mGluR-dependent long-term depression is associated with increased phosphorylation of S6 and synthesis of elongation factor 1A but remains expressed in S6K-deficient mice. Mol. Cell. Biol. 28, 2996–3007 10.1128/MCB.00201-08 18316404 PMC2293080

[B2] Banko J. L. Hou L. Poulin F. Sonenberg N. Klann E. (2006). Regulation of eukaryotic initiation factor 4E by converging signaling pathways during metabotropic glutamate receptor-dependent long-term depression. J. Neurosci. 26, 2167–2173 10.1523/JNEUROSCI.5196-05.2006 16495443 PMC6674817

[B3] Bateup H. S. Johnson C. A. Denefrio C. L. Saulnier J. L. Kornacker K. Sabatini B. L. (2013). Excitatory/inhibitory synaptic imbalance leads to hippocampal hyperexcitability in mouse models of tuberous sclerosis. Neuron 78, 510–522 10.1016/j.neuron.2013.03.017 23664616 PMC3690324

[B4] Bateup H. S. Takasaki K. T. Saulnier J. L. Denefrio C. L. Sabatini B. L. (2011). Loss of Tsc1 *in vivo* impairs hippocampal mGluR-LTD and increases excitatory synaptic function. J. Neurosci. 31, 8862–8869 10.1523/JNEUROSCI.1617-11.2011 21677170 PMC3133739

[B5] Beique J. C. Na Y. Kuhl D. Worley P. F. Huganir R. L. (2011). Arc-dependent synapse-specific homeostatic plasticity. Proc. Natl. Acad. Sci. U.S.A. 108, 816–821 10.1073/pnas.1017914108 21187403 PMC3021034

[B6] Bramham C. R. Alme M. N. Bittins M. Kuipers S. D. Nair R. R. Pai B. (2010). The Arc of synaptic memory. Exp. Brain Res. 200, 125–140 10.1007/s00221-009-1959-2 19690847 PMC2803749

[B7] Briz V. Hsu Y. T. Li Y. Lee E. Bi X. Baudry M. (2013). Calpain-2-mediated PTEN degradation contributes to BDNF-induced stimulation of dendritic protein synthesis. J. Neurosci. 33, 4317–4328 10.1523/JNEUROSCI.4907-12.2013 23467348 PMC3657575

[B8] Chowdhury S. Shepherd J. D. Okuno H. Lyford G. Petralia R. S. Plath N. (2006). Arc/Arg3.1 interacts with the endocytic machinery to regulate AMPA receptor trafficking. Neuron 52, 445–459 10.1016/j.neuron.2006.08.033 17088211 PMC1784006

[B9] Costa-Mattioli M. Sossin W. S. Klann E. Sonenberg N. (2009). Translational control of long-lasting synaptic plasticity and memory. Neuron 61, 10–26 10.1016/j.neuron.2008.10.055 19146809 PMC5154738

[B10] Davis G. W. (2006). Homeostatic control of neural activity: from phenomenology to molecular design. Annu. Rev. Neurosci. 29, 307–323 10.1146/annurev.neuro.28.061604.135751 16776588

[B11] Fu A. K. Hung K. W. Fu W. Y. Shen C. Chen Y. Xia J. (2011). APC (Cdh1) mediates EphA4-dependent downregulation of AMPA receptors in homeostatic plasticity. Nat. Neurosci. 14, 181–189 10.1038/nn.2715 21186356

[B12] Gallagher S. M. Daly C. A. Bear M. F. Huber K. M. (2004). Extracellular signal-regulated protein kinase activation is required for metabotropic glutamate receptor-dependent long-term depression in hippocampal area CA1. J. Neurosci. 24, 4859–4864 10.1523/JNEUROSCI.5407-03.2004 15152046 PMC6729463

[B13] Gelinas J. N. Banko J. L. Hou L. Sonenberg N. Weeber E. J. Klann E. (2007). ERK and mTOR signaling couple beta-adrenergic receptors to translation initiation machinery to gate induction of protein synthesis-dependent long-term potentiation. J. Biol. Chem. 282, 27527–27535 10.1074/jbc.M701077200 17635924

[B14] Giorgi C. Yeo G. W. Stone M. E. Katz D. B. Burge C. Turrigiano G. (2007). The EJC factor eIF4AIII modulates synaptic strength and neuronal protein expression. Cell 130, 179–191 10.1016/j.cell.2007.05.028 17632064

[B15] Goold C. P. Nicoll R. A. (2010). Single-cell optogenetic excitation drives homeostatic synaptic depression. Neuron 68, 512–528 10.1016/j.neuron.2010.09.020 21040851 PMC3111089

[B16] Greer P. L. Greenberg M. E. (2008). From synapse to nucleus: calcium-dependent gene transcription in the control of synapse development and function. Neuron 59, 846–860 10.1016/j.neuron.2008.09.002 18817726

[B17] Greer P. L. Hanayama R. Bloodgood B. L. Mardinly A. R. Lipton D. M. Flavell S. W. (2010). The Angelman Syndrome protein Ube3A regulates synapse development by ubiquitinating Arc. Cell 140, 704–716 10.1016/j.cell.2010.01.026 20211139 PMC2843143

[B18] Hou L. Klann E. (2004). Activation of the phosphoinositide 3-kinase-Akt-mammalian target of rapamycin signaling pathway is required for metabotropic glutamate receptor-dependent long-term depression. J. Neurosci. 24, 6352–6361 10.1523/JNEUROSCI.0995-04.2004 15254091 PMC6729543

[B19] Huber K. M. Gallagher S. M. Warren S. T. Bear M. F. (2002). Altered synaptic plasticity in a mouse model of fragile X mental retardation. Proc. Natl. Acad. Sci. U.S.A. 99, 7746–7750 10.1073/pnas.122205699 12032354 PMC124340

[B20] Kawashima T. Okuno H. Nonaka M. Adachi-Morishima A. Kyo N. Okamura M. (2009). Synaptic activity-responsive element in the Arc/Arg3.1 promoter essential for synapse-to-nucleus signaling in activated neurons. Proc. Natl. Acad. Sci. U.S.A. 106, 316–321 10.1073/pnas.0806518106 19116276 PMC2629236

[B21] Kelleher R. J. 3rd. Govindarajan A. Jung H. Y. Kang H. Tonegawa S. (2004). Translational control by MAPK signaling in long-term synaptic plasticity and memory. Cell 116, 467–479 10.1016/S0092-8674(04)00115-1 15016380

[B22] Korb E. Wilkinson C. L. Delgado R. N. Lovero K. L. Finkbeiner S. (2013). Arc in the nucleus regulates PML-dependent GluA1 transcription and homeostatic plasticity. Nat. Neurosci. 16, 874–883 10.1038/nn.3429 23749147 PMC3703835

[B23] Kwiatkowski D. J. Zhang H. Bandura J. L. Heiberger K. M. Glogauer M. El-Hashemite N. (2002). A mouse model of TSC1 reveals sex-dependent lethality from liver hemangiomas, and up-regulation of p70S6 kinase activity in Tsc1 null cells. Hum. Mol. Genet. 11, 525–534 10.1093/hmg/11.5.525 11875047

[B24] Link W. Konietzko U. Kauselmann G. Krug M. Schwanke B. Frey U. (1995). Somatodendritic expression of an immediate early gene is regulated by synaptic activity. Proc. Natl. Acad. Sci. U.S.A. 92, 5734–5738 10.1073/pnas.92.12.5734 7777577 PMC41771

[B25] Luscher C. Huber K. M. (2010). Group 1 mGluR-dependent synaptic long-term depression: mechanisms and implications for circuitry and disease. Neuron 65, 445–459 10.1016/j.neuron.2010.01.016 20188650 PMC2841961

[B26] Lyford G. L. Yamagata K. Kaufmann W. E. Barnes C. A. Sanders L. K. Copeland N. G. (1995). Arc, a growth factor and activity-regulated gene, encodes a novel cytoskeleton-associated protein that is enriched in neuronal dendrites. Neuron 14, 433–445 10.1016/0896-6273(95)90299-6 7857651

[B27] Ma L. Chen Z. Erdjument-Bromage H. Tempst P. Pandolfi P. P. (2005). Phosphorylation and functional inactivation of TSC2 by Erk implications for tuberous sclerosis and cancer pathogenesis. Cell 121, 179–193 10.1016/j.cell.2005.02.031 15851026

[B29] Ma X. M. Blenis J. (2009). Molecular mechanisms of mTOR-mediated translational control. Nat. Rev. Mol. Cell Biol. 10, 307–318 10.1038/nrm2672 19339977

[B28] Marder E. Goaillard J. M. (2006). Variability, compensation, and homeostasis in neuron and network function. Nat. Rev. Neurosci. 7, 563–574 10.1038/nrn1949 16791145

[B30] Mendoza M. C. Er E. E. Blenis J. (2011). The Ras-ERK and PI3K-mTOR pathways: cross-talk and compensation. Trends Biochem. Sci. 36, 320–328 10.1016/j.tibs.2011.03.006 21531565 PMC3112285

[B31] Messaoudi E. Kanhema T. Soule J. Tiron A. Dagyte G. Da Silva B. (2007). Sustained Arc/Arg3.1 synthesis controls long-term potentiation consolidation through regulation of local actin polymerization in the dentate gyrus *in vivo*. J. Neurosci. 27, 10445–10455 10.1523/JNEUROSCI.2883-07.2007 17898216 PMC6673172

[B32] Panja D. Dagyte G. Bidinosti M. Wibrand K. Kristiansen A. M. Sonenberg N. (2009). Novel translational control in Arc-dependent long term potentiation consolidation *in vivo*. J. Biol. Chem. 284, 31498–31511 10.1074/jbc.M109.056077 19755425 PMC2797219

[B33] Park S. Park J. M. Kim S. Kim J. A. Shepherd J. D. Smith-Hicks C. L. (2008). Elongation factor 2 and fragile X mental retardation protein control the dynamic translation of Arc/Arg3.1 essential for mGluR-LTD. Neuron 59, 70–83 10.1016/j.neuron.2008.05.023 18614030 PMC2743934

[B34] Peebles C. L. Yoo J. Thwin M. T. Palop J. J. Noebels J. L. Finkbeiner S. (2010). Arc regulates spine morphology and maintains network stability *in vivo*. Proc. Natl. Acad. Sci. U.S.A. 107, 18173–18178 10.1073/pnas.1006546107 20921410 PMC2964216

[B35] Perkinton M. S. Sihra T. S. Williams R. J. (1999). Ca(2+)-permeable AMPA receptors induce phosphorylation of cAMP response element-binding protein through a phosphatidylinositol 3-kinase-dependent stimulation of the mitogen-activated protein kinase signaling cascade in neurons. J. Neurosci. 19, 5861–5874 10407026 10.1523/JNEUROSCI.19-14-05861.1999 PMC6783096

[B36] Pintchovski S. A. Peebles C. L. Kim H. J. Verdin E. Finkbeiner S. (2009). The serum response factor and a putative novel transcription factor regulate expression of the immediate-early gene Arc/Arg3.1 in neurons. J. Neurosci. 29, 1525–1537 10.1523/JNEUROSCI.5575-08.2009 19193899 PMC2874324

[B37] Pozo K. Goda Y. (2010). Unraveling mechanisms of homeostatic synaptic plasticity. Neuron 66, 337–351 10.1016/j.neuron.2010.04.028 20471348 PMC3021747

[B38] Rao V. R. Pintchovski S. A. Chin J. Peebles C. L. Mitra S. Finkbeiner S. (2006). AMPA receptors regulate transcription of the plasticity-related immediate-early gene Arc. Nat. Neurosci. 9, 887–895 10.1038/nn1708 16732277

[B39] Rial Verde E. M. Lee-Osbourne J. Worley P. F. Malinow R. Cline H. T. (2006). Increased expression of the immediate-early gene Arc/Arg3.1 reduces AMPA receptor-mediated synaptic transmission. Neuron 52, 461–474 10.1016/j.neuron.2006.09.031 17088212 PMC3951199

[B40] Saha R. N. Wissink E. M. Bailey E. R. Zhao M. Fargo D. C. Hwang J. Y. (2011). Rapid activity-induced transcription of Arc and other IEGs relies on poised RNA polymerase II. Nat. Neurosci. 14, 848–856 10.1038/nn.2839 21623364 PMC3125443

[B41] Shepherd J. D. Bear M. F. (2011). New views of Arc, a master regulator of synaptic plasticity. Nat. Neurosci. 14, 279–284 10.1038/nn.2708 21278731 PMC8040377

[B42] Shepherd J. D. Rumbaugh G. Wu J. Chowdhury S. Plath N. Kuhl D. (2006). Arc/Arg3.1 mediates homeostatic synaptic scaling of AMPA receptors. Neuron 52, 475–484 10.1016/j.neuron.2006.08.034 17088213 PMC1764219

[B43] Soule J. Alme M. Myrum C. Schubert M. Kanhema T. Bramham C. R. (2012). Balancing Arc synthesis, mRNA decay, and proteasomal degradation: maximal protein expression triggered by rapid eye movement sleep-like bursts of muscarinic cholinergic receptor stimulation. J. Biol. Chem. 287, 22354–22366 10.1074/jbc.M112.376491 22584581 PMC3381195

[B44] Takei N. Inamura N. Kawamura M. Namba H. Hara K. Yonezawa K. (2004). Brain-derived neurotrophic factor induces mammalian target of rapamycin-dependent local activation of translation machinery and protein synthesis in neuronal dendrites. J. Neurosci. 24, 9760–9769 10.1523/JNEUROSCI.1427-04.2004 15525761 PMC6730227

[B45] Takeuchi K. Gertner M. J. Zhou J. Parada L. F. Bennett M. V. Zukin R. S. (2013). Dysregulation of synaptic plasticity precedes appearance of morphological defects in a Pten conditional knockout mouse model of autism. Proc. Natl. Acad. Sci. U.S.A. 110, 4738–4743 10.1073/pnas.1222803110 23487788 PMC3607034

[B46] Tian X. Feig L. A. (2006). Age-dependent participation of Ras-GRF proteins in coupling calcium-permeable AMPA glutamate receptors to Ras/Erk signaling in cortical neurons. J. Biol. Chem. 281, 7578–7582 10.1074/jbc.M512060200 16407208

[B47] Toro R. Konyukh M. Delorme R. Leblond C. Chaste P. Fauchereau F. (2010). Key role for gene dosage and synaptic homeostasis in autism spectrum disorders. Trends Genet. 26, 363–372 10.1016/j.tig.2010.05.007 20609491

[B48] Turrigiano G. (2012). Homeostatic synaptic plasticity: local and global mechanisms for stabilizing neuronal function. Cold Spring Harb. Perspect. Biol. 4:a005736 10.1101/cshperspect.a005736 22086977 PMC3249629

[B49] Turrigiano G. G. Leslie K. R. Desai N. S. Rutherford L. C. Nelson S. B. (1998). Activity-dependent scaling of quantal amplitude in neocortical neurons. Nature 391, 892–896 10.1038/36103 9495341

[B50] Watt A. J. Van Rossum M. C. Macleod K. M. Nelson S. B. Turrigiano G. G. (2000). Activity coregulates quantal AMPA and NMDA currents at neocortical synapses. Neuron 26, 659–670 10.1016/S0896-6273(00)81202-7 10896161

[B51] Waung M. W. Pfeiffer B. E. Nosyreva E. D. Ronesi J. A. Huber K. M. (2008). Rapid translation of Arc/Arg3.1 selectively mediates mGluR-dependent LTD through persistent increases in AMPAR endocytosis rate. Neuron 59, 84–97 10.1016/j.neuron.2008.05.014 18614031 PMC2580055

[B52] Wiegert J. S. Bading H. (2011). Activity-dependent calcium signaling and ERK-MAP kinases in neurons: a link to structural plasticity of the nucleus and gene transcription regulation. Cell Calcium 49, 296–305 10.1016/j.ceca.2010.11.009 21163523

[B53] Ying S. W. Futter M. Rosenblum K. Webber M. J. Hunt S. P. Bliss T. V. (2002). Brain-derived neurotrophic factor induces long-term potentiation in intact adult hippocampus: requirement for ERK activation coupled to CREB and upregulation of Arc synthesis. J. Neurosci. 22, 1532–1540 11880483 10.1523/JNEUROSCI.22-05-01532.2002 PMC6758896

[B54] Yuan L. L. Chen X. Kunjilwar K. Pfaffinger P. Johnston D. (2006). Acceleration of K+ channel inactivation by MEK inhibitor U0126. Am. J. Physiol. Cell Physiol. 290, C165–C171 10.1152/ajpcell.00206.2005 16135544

[B55] Zoghbi H. Y. Bear M. F. (2012). Synaptic dysfunction in neurodevelopmental disorders associated with autism and intellectual disabilities. Cold Spring Harb. Perspect. Biol. 4, 1–22 10.1101/cshperspect.a009886 22258914 PMC3282414

